# Online e-Cigarette Retailers’ Use of Price Incentives and Product Features to Attract Adolescent and Young Adult Purchases: Cross-Sectional Choice-Based Study

**DOI:** 10.2196/75128

**Published:** 2025-09-29

**Authors:** Shivani Mathur Gaiha, Catherine Stamoulis

**Affiliations:** 1 Division of Adolescent/Young Adult Medicine, Department of Pediatrics, Boston Children's Hospital, 1 Autumn St, 5th Floor, Boston, MA, 02115, United States, 1 617-355-3538; 2 Department of Pediatrics, Harvard Medical School, Boston, MA, United States

**Keywords:** e-cigarettes, internet, nicotine, regulatory science, marketing, advertising and promotion, adolescents and young adults, choice-based experiment, maximum difference

## Abstract

**Background:**

There are reports of several adolescents and young adults purchasing electronic cigarettes (e-cigarettes) from e-cigarette retailer websites. However, there is a lack of consumer research on how e-cigarette retailer websites’ content influences adolescent and young adult purchases.

**Objective:**

This study aims to examine whether seeing specific characteristics on e-cigarette retailer websites encourages or discourages e-cigarette purchase by adolescents and young adults and to further assess whether such influences vary by age and e-cigarette use.

**Methods:**

Using a web-based survey, we conducted a cross-sectional choice-based experiment in 5326 individuals (aged 13‐24 years). We examined associations between e-cigarette retailer websites’ content, reflected in 18 keywords, and the likelihood of e-cigarette purchase.

**Results:**

Female and sexual minority (nonheterosexual) adolescents and young adults were more likely to purchase e-cigarettes if websites had a clearance or sale (female individuals: adjusted odds ratio [aOR] 1.19, 95% CI 1.04‐1.35; *P=*.02; sexual minority individuals: aOR 1.48, 95% CI 1.21‐1.82; *P<*.01), deals (sexual minority individuals: aOR 1.28, 95% CI 1.04‐1.57; *P=*.02), or direct discounts (female individuals: aOR 1.26, 95% CI 1.09‐1.45; *P<*.01; sexual minority individuals: aOR 1.29, 95% CI 1.03‐1.61; *P=*.03); if they could shop by flavor (female individuals: aOR 1.49, 95% CI 1.30‐1.71; *P<*.01); or if they saw new and trending products (female individuals: aOR 1.18, 95% CI 1.03‐1.34; *P=*.03). Female individuals were less likely to purchase e-cigarettes if required to register or sign-in (aOR 0.66, 95% CI 0.58‐0.76; *P<*.01) or upload an ID (aOR 0.84, 95% CI 0.74‐0.96; *P=*.02), and similarly, sexual minority individuals were less likely to purchase e-cigarettes if required to register or sign-in (aOR 0.77, 95% CI 0.63‐0.96; *P*=.02). Older participants were more likely to purchase from authorized dealers (aOR 1.06, 95% CI 1.03‐1.08; *P*<.01) if required to enter an email (aOR 1.03, 95% CI 1.01‐1.06; *P*=.01) or a photo ID (aOR 1.11, 95% CI 1.08‐1.13; *P<*.01) but less likely to purchase based on seeing new and trending products (aOR 0.94, 95% CI 0.92‐0.96; *P<*.01) or vape guides or blogs (aOR 0.97, 95% CI 0.95‐0.99; *P=*.04). Participants who had never used e-cigarettes were more susceptible to purchase if they saw a starter kit (aOR 1.27, 95% CI 1.07‐1.51; *P=*.01), if they saw new and trending products (aOR 1.28, 95% CI 1.08‐1.52; *P<*.01), or if they could shop by flavor (aOR 1.67, 95% CI 1.39‐2.00; *P<*.01).

**Conclusions:**

The findings show that adolescents and young adults are attracted to several aspects of e-cigarette retailer website content. Providing tailored prevention education about price incentives, flavors, and starter kits to vulnerable groups at risk of purchase and ensuring effective age verification could discourage e-cigarette online purchases.

## Introduction

Adolescents and young adults have easy access to nicotine electronic cigarettes (e-cigarettes), often making purchases online through e-cigarette retailer websites. These websites may be selling single- or multibrand e-cigarettes and may be owned and operated by e-cigarette companies, dealers, or retail stores. At present, the Food and Drug Administration (FDA) requires all websites marketing e-cigarettes to include an explicit nicotine warning label to inform all web users that e-cigarettes are addictive [1] and are only being marketed to those above the age of 21 years [2]. However, adolescents and young adults are exposed to multiple other e-cigarette marketing characteristics on these web pages that could potentially influence their decision to purchase e-cigarettes. Since e-cigarette–related information seeking is associated with e-cigarette use [3], identifying the attractive characteristics of these websites could help in developing prevention education for adolescents and young adults.

e-Cigarette retailer websites’ content is an understudied area. Prior research shows that flavors, such as fruit and dessert; brands representing perceived value and trendiness; and affordability are features of e-cigarette products that are generally appealing to adolescents and young adults [4-8]. Researcher-led content analyses of social media and e-cigarette retailer websites have identified a range of appealing characteristics, including flavors, brands, “starter-kit” labels, price discounts including coupons and sales, and ability of users to verify their age [9-15]. However, it is unclear which online marketing characteristics are likely to be found influencing adolescents and young adults in purchasing e-cigarettes from e-cigarette retailer websites. Although content and keyword restrictions are not feasible, evidence of marketing characteristics that are important to adolescents and young adults, especially the younger ones and those who currently use or have never used e-cigarettes, can help identify specific adolescent and young adult groups that may be more susceptible to online e-cigarette marketing strategies. Furthermore, data on the effects of inconsistently applied age verification methods [12,13] may help our understanding in developing methods to discourage e-cigarette online purchases.

To address this knowledge gap, this study conducted a maximum difference experiment [16] to address the following questions:

1. What specific characteristics on e-cigarette retailer websites are most influential in encouraging and discouraging e-cigarette purchase by adolescents and young adults? Do they vary by age and by e-cigarette use status?
2. Are never-users who are susceptible to using e-cigarettes more likely to purchase them if they see specific characteristics on e-cigarette retailer websites?

## Methods

### Study Design and Participants

We conducted a web-based, self-administered cross-sectional survey among adolescents and young adults aged 13 to 24 years in the United States who were recruited through a convenience sample. Survey data were collected from December 12, 2023, to March 13, 2024. Participants were recruited through Qualtrics’ online nonprobability panels to match sex at birth, race, and ethnicity per the US Census through sampling quotas. Panel providers sent out a study link to prospective participants, and those who clicked the link and provided consent or assent were included in the study. After initial recruitment (till February 2024), during which we aimed to recruit equal proportions of participants aged 13 to 17 and 18 to 24 years, we observed that fewer adolescents (aged 13-17 years) entered the study and did not enforce a targeted recruitment to increase the representation of this age range in the sample. Participants were included irrespective of previous e-cigarette use. We excluded participants who submitted the survey in a median of <8.45 minutes, which was half the median time taken to complete the survey. We reported the results per the Checklist for Reporting Results of Internet E-Surveys (CHERRIES) guidelines.

### Ethical Considerations

This study was approved by the institutional review board at Stanford University (54761). Participants anonymously completed surveys including consent/assent documentation, and no identifiable personal health information was collected. All participants provided voluntary assent; individuals aged below 18 years provided assent (waiver of parental consent), and participants above the age of 18 years provided consent to participate in this study. Participants received compensation up to US $5 in a form (eg, video game tokens) corresponding to their prior agreement with their panel provider.

### Measures

Participants provided sociodemographic data, including age, sex assigned at birth, race, ethnicity, education level, gender, and sexual orientation. Gender identities included woman, man, nonbinary, another gender, and prefer not to say. Due to small samples in some categories, gender identity was represented in statistical models by a 3-category variable: man, woman, or other. Similarly, sexual orientation (originally categorized as heterosexual or straight, bisexual, gay, lesbian, asexual, or pansexual) was included in models as heterosexual or straight, bisexual, or other. Finally, race and ethnicity were combined into a single categorical variable that included White non-Hispanic, Black non-Hispanic, Asian or Hawaiian or Pacific Islander non-Hispanic, other or multiracial non-Hispanic, and Hispanic. In statistical analyses, it was represented by a 4-category variable: White non-Hispanic, Black non-Hispanic, other non-Hispanic, and Hispanic.

### e-Cigarette Use

We asked participants whether they had ever used a nicotine e-cigarette, even one or two puffs, with the following response categories: (1) yes, in the past 30 days; (2) yes, but not in the past 30 days; and (3) no. In addition, for those who had never vaped, we assessed their susceptibility to e-cigarette use (adapted from the Enhanced Susceptibility Index) [17] as a binary (yes or no) variable, which was estimated from answers to a 4-point Likert scale on whether they were curious about vaping using an e-cigarette (scored as 1=not at all curious, 2=a little curious, 3=somewhat curious, and 4=very curious). We also asked participants how likely they were to use e-cigarettes if a friend offered them and if they were planning to try e-cigarettes in the next month, 6 months, or a year (scored as 1=definitely will not, 2=probably will not, 3=possibly will, and 4=definitely will use or try). Participants with a score >5 were considered susceptible to e-cigarette use.

### Maximum Difference Experiment to Assess the Likelihood of Purchase on Viewing Characteristics on an e-Cigarette Retailer Website

Participants were presented with a maximum difference choice task [16] to view and determine the influence of 18 keywords on their decision to purchase e-cigarettes.

We showed participants a screenshot of an existing website for EBCreate (a nicotine e-cigarette brand), asking them to think of similar single- or multibrand websites marketing e-cigarettes. Next, we asked participants, “Which of the following words on a nicotine vape website (ie, Elfbar.com/EBCreate.com or Vuse.com or Vapor.com) would make it more likely or less likely for you to purchase a nicotine vape/e-cigarette?” (Figure S1 in Multimedia Appendix 1). Nine sets of 4 randomly selected keywords (out of a total of 18 keywords) were presented sequentially. Therefore, some participants saw some keywords more than once during the experiment. The keywords were selected based on a literature review and qualitative research with adolescents and young adults [18,19] and represented the following 5 themes:

1. Product features: “shop by flavor,” “shop top sellers,” “shop by bottle size,” “shop by brand,” and “shop by color”
2. Price incentives: “clearance or sale,” “deals (promo code, buy-one-get-one free, and multipack),” and “direct discount (eg, 15% off)”
3. Product curation: “new and trending,” “specs and features,” “bestsellers,” and “starter kit”
4. Product rating or standard: “authorized dealer,” “vape guides or blogs (eg, Best of 2023),” and “customer ratings or reviews”
5. Age verification: “enter email,” “register or sign-in (each time),” and “upload photo ID”

Participants could choose one or more keywords as “more likely” or “less likely” or not make a choice. There was no back button.

### Statistical Analyses

All analyses were performed using 2 levels of statistical modeling. First, to identify a parsimonious set of independent variables that were associated with each keyword, we used shrinkage regression (based on the least absolute shrinkage and selection operator) [20]. Given that this dimensionality reduction approach minimizes overfitting and may thus exclude variables that are important to address our hypotheses, we also used least absolute shrinkage and selection operator–selected variables as the basis for larger models that were augmented with additional parameters (using stepwise model augmentation). Participant age and e-cigarette use were included in all models. Since some participants saw keywords more than once during the experiment, mixed effects regression models were developed and included a random intercept and slope for participants.

The first set of models included all participants with the status as never used e-cigarettes, used e-cigarettes but not in the past 30 days, and used an e-cigarette in the past 30 days. The second set compared those who had never used an e-cigarette to those who had used in the past 30 days. The third set only included participants who had never used e-cigarettes and assessed their susceptibility to use. Separate models examined (1) associations between susceptibility and online content, that is, each presented keyword (adjusting for participant characteristics), and (2) associations between susceptibility and participant characteristics independently of their responses to keywords.

A number of participants had inconsistent responses to some keywords, choosing “less likely” at one presentation and “more likely” in the other. Across analyses, the primary results are based on samples that included only participants with consistent responses. Additional results based on models that included all participants regardless of the consistency of their repeated responses are reported in Tables S1-S3 in Multimedia Appendix 1. Across models, parameter *P* values were corrected for the false discovery rate, for all the 18 items [21]. Data were analyzed using Matlab (release R2024b; Mathworks, Inc).

## Results

A cohort of 5326 adolescents and young adults who were aged 13 to 24 years (female individuals assigned at birth: 3013/5326, 56.6%) was included in the study. The median age was 18 (IQR 16-21) years. In total, 2308 (43.33%) participants identified as women, 608 (11.42%) were non-Hispanic Black, 1055 (19.81%) were Hispanic, and 3207 (60.21%) were non-Hispanic White. Less than half of the participants had completed high school (2269/5326, 42.60%). e-Cigarette use status was as follows: 55.16% (2938/5326) had never used an e-cigarette, 18.21% (970/5326) had used an e-cigarette but not in the past 30 days, and 26.62% (1418/5326) had used an e-cigarette in the past 30 days. The median (IQR) age at first vape was 13 (13-22) years (Table 1). Almost half of those who had never used e-cigarettes were susceptible to use (1352/5326, 46.02%). Multiple factors influenced adolescents and young adults to browse online including social media and advertisements promoting e-cigarettes (1107/5326, 20.78%), family and friends’ influence (725/5326, 13.61%), feeling bored (603/5326, 11.32%), or feeling stressed (515/5326, 9.67%).

Participant characteristics are provided in Table 2.

The distribution of factors likely to influence e-cigarette purchase is shown in Table 2. Deals, direct discounts, and clearance were the most commonly identified keywords likely to encourage e-cigarette purchase on e-cigarette retailer websites. In contrast, age verification, including uploading a photo ID, registering or signing-in each time, and entering an email, was least likely to encourage purchase.

In the sample that included all participants with consistent responses to each keyword, female individuals were more likely to purchase e-cigarettes if websites had clearance or sales, a direct discount, option for shopping by flavor, or product novelty or trendiness (adjusted odds ratio [aOR] 1.18-1.49, 95% CI 1.03-1.71; *P*≤.03) and less likely to purchase if they had to register or sign-in each time or upload an ID (aOR 0.66‐0.84, 95% CI 0.58‐0.96; *P*≤.02). Similarly, sexual minority individuals (nonheterosexual) were more likely to purchase if the website offered price incentives such as deals or a direct discount or had a clearance or sale (aOR 1.28‐1.48, 95% CI 1.04‐1.82; *P*≤.02) and less likely to purchase if each time they had to register or sign-in (aOR 0.77, 95% CI 0.63‐0.96; *P*=.02). Older participants were more likely to purchase e-cigarettes from authorized dealers or websites where they had to enter their email or upload their ID (aOR 1.03‐1.11, 95% CI 1.01‐1.13; *P*≤.01) and less likely to purchase based on new and trending products or vape guides or blogs (aOR 0.94‐0.97, 95% CI 0.92‐0.99; *P*≤.04). Finally, those who had ever used an e-cigarette were more likely to purchase if they had the option to shop by flavor (aOR 1.31, 95% CI 1.20‐1.44; *P*<.01) but less likely to purchase if the website offered a starter kit or they had to enter an email ID or upload a photo ID (aOR 0.85‐0.88, 95% CI 0.78‐0.97; *P*≤.02). Detailed model statistics are provided in Table 3 . Similar associations were estimated in the larger sample that included participants with inconsistent responses to each keyword during the maximum difference experiment (Table S1 in Multimedia Appendix 1).

**Table 1.** Demographic and other participant characteristics.

Characteristics	Participants
Age (y), median (IQR)	18 (16‐21)
Sex assigned at birth, n (%)	
Male	2219 (41.7)
Female	3013 (56.6)
Missing (including prefer not to say)	94 (1.7)
Gender, n (%)	
Man	2779 (52.2)
Woman	2308 (43.3)
Nonbinary	149 (2.8)
Another gender	31 (0.6)
Missing (including prefer not to say)	59 (1.1)
Sexual orientation, n (%)	
Heterosexual or straight	3341 (62.7)
Bisexual	728 (13.7)
Asexual	270 (5.1)
Gay	144 (2.7)
Lesbian	125 (2.3)
Pansexual	180 (3.4)
Missing	538 (10.1)
Race or ethnicity, n (%)	
Asian or Hawaiian or Pacific Islander non-Hispanic	214 (4.0)
Black non-Hispanic	608 (11.4)
Hispanic	1055 (19.8)
White non-Hispanic	3207 (60.2)
Other or multiracial	41 (0.8)
Missing both race and ethnicity	201 (3.8)
Education, n (%)	
Elementary or less	92 (1.7)
Middle school or junior high	1503 (28.2)
High school	2269 (42.6)
Some college	735 (13.8)
2-year (associate) degree	247 (4.7)
4-year college degree	314 (5.9)
Advance degree (master’s, doctorate, or professional)	162 (3.1)
Missing	4 (<0.1)
e-Cigarette use, n (%)	
Never	2938 (55.2)
Yes, but not in the last 30 days	970 (18.2)
Yes, in the last 30 days	1418 (26.6)
Age at first vape (y), median (IQR)	13 (13-22)

**Table 2.** Distribution of factors that are most likely and least likely to influence e-cigarette purchase^a^.

Items	Total responses (including repeated), n	More likely, n (%)	Less likely, n (%)
Authorized dealer	5222	2789 (53.4)	2433 (46.6)
Bestsellers	4964	3362 (67.7)	1602 (32.3)
Clearance or sale	5813	3995 (68.7)	1818 (31.3)
Customer rating (≥3)	5045	2443 (48.4)	2602 (51.6)
Deals (promo code, BOGOF^b^, or multipack)	6192	4381 (70.8)	1811 (29.2)
Direct discount	5786	4225 (73.0)	1561 (27.0)
Enter email	5957	1446 (24.3)	4511 (75.7)
New and trending	5042	2870 (56.9)	2172 (43.1)
Register or sign-in (each time)	6179	1508 (24.4)	4671 (75.6)
Shop top sellers	4652	2722 (58.5)	1930 (41.5)
Shop by bottle size	4493	1767 (39.3)	2726 (60.7)
Shop by brand	4490	2361 (52.6)	2129 (47.4)
Shop by color	4906	2052 (41.8)	2854 (58.2)
Shop by flavor	5234	3566 (68.1)	1668 (31.9)
Specs and features	4456	2079 (46.7)	2377 (53.3)
Starter kit	4814	2506 (52.1)	2308 (47.9)
Upload photo ID	7259	1741 (24.0)	5518 (76.0)
Vape guide or blog (eg, Best of 2023)	5310	2094 (39.4)	3216 (60.6)

a Numbers are out of the total number of responses that include repeated responses if participants saw a factor more than once during the experiment.

b BOGOF: buy-one-get-one free.

**Table 3.** Statistics of models testing associations between participant characteristics and e-cigarette use and the likelihood of purchasing based on individual marketing factors. Results are based on a sample with consistent repeated responses and any e-cigarette use status.

Items and parameters		aOR^a^ (95% CI)		*P* value^b^
Product features				
Shop by flavor				
e-Cigarette use		1.31 (1.20-1.44)		<.01
Female		1.49 (1.30-1.71)		<.01
Shop top sellers				
NS^c^		—^d^		—
Shop by bottle size				
NS		—		—
Shop by brand				
NS		—		—
Shop by color				
NS		—		—
Price incentives				
Clearance or sale				
Female		1.19 (1.04-1.35)		.02
Sexual minority individual		1.48 (1.21-1.82)		<.01
Deals (promo code, BOGOF^e^, and multipack)				
Sexual minority individual		1.28 (1.04-1.57)		.02
Direct discount				
Female		1.26 (1.09-1.45)		<.01
Sexual minority individual		1.29 (1.03-1.61)		.03
Product curation				
New and trending				
Age (y)		0.9 (0.92-0.96)		<.01
Female		1.18 (1.03-1.34)		.03
Specs and features				
NS		—		—
Bestsellers				
NS		—		—
Starter kit				
e-Cigarette use		0.87 (0.80-0.95)		<.01
Product rating or standard				
Authorized dealer				
Age (y)		1.06 (1.03-1.08)		<.01
Vape guide or blog (eg, Best of 2023)				
Age (y)		0.97 (0.95-0.99)		.04
Customer rating (≥3)				
NS		—		—
Age verification				
Enter email				
Age (y)		1.03 (1.01-1.06)		.01
e-Cigarette use		0.88 (0.81-0.97)		.02
Register or sign-in (each time)				
Female		0.66 (0.58-0.76)		<.01
Sexual minority individual		0.77 (0.63-0.96)		.02
Upload photo ID				
Age (y)		1.11 (1.08-1.13)		<.01
e-Cigarette use		0.85 (0.78-0.92)		.01
Female		0.84 (0.74-0.96)		.02

a aOR: adjusted odds ratio.

b All reported *P* values were corrected for the false discovery rate (FDR), over the 18 items.

c NS: not significant:

d Not applicable.

e BOGOF: buy-one-get-one free.

In the sample that compared participants who had never used e-cigarettes to those who had used in the last 30 days, similar associations were identified. Female individuals were more likely to purchase e-cigarettes based on bestsellers, clearance or sale, deals, direct discounts, option to shop by flavor (aOR 1.19‐1.56, 95% CI 1.02‐1.82; *P*≤.04), and less likely to purchase if they needed to register or sign-in each time or upload their ID (aOR 0.65‐0.83, 95% CI 0.56‐0.95; *P*≤.02). Sexual minority individuals were more likely to purchase based on clearance or sale and option to shop by flavor (aOR 1.40‐1.59, 95% CI 1.12‐2.06; *P*<.01). Older participants were more likely to purchase from an authorized dealer and websites that required them to enter their email, register or sign-in each time, or upload their ID (aOR 1.05‐1.11, 95% CI 1.02‐1.13; *P*≤.01). In contrast, they were less likely to purchase based on a clearance or sale, new and trending products, option to shop by flavor, or availability of a starter kit (aOR 0.93‐0.97, 95% CI 0.90‐0.99; *P*≤.02). Compared to those who had never used e-cigarettes, those who used e-cigarettes in the past 30 days were more likely to purchase e-cigarettes based on an option to shop by flavor (aOR 1.32, 95% CI 1.20‐1.45; *P*<.01) but less likely to do so based on the availability of a starter kit or requirement to enter an email ID or upload a photo ID (aOR 0.87‐0.89, 95% CI 0.80‐0.98; *P*≤.04). Detailed model statistics are provided in Table 4. Similar associations were estimated in the larger sample that included participants with inconsistent responses to the keywords (Table S2 in Multimedia Appendix 1).

**Table 4.** Statistics of models testing associations between participant characteristics and e-cigarette use and the likelihood of purchasing based on individual marketing factors. Results are based on a sample with consistent, repeated responses and included only those who had never vaped and those who had vaped in the past 30 days.

Items and parameters		aOR^a^ (95% CI)		*P* value^b^
Product features				
Shop by flavor				
Age (y)		0.96 (0.94-0.98)		<.01
e-Cigarette use		1.32 (1.20-1.45)		<.01
Female		1.56 (1.34-1.82)		<.01
Sexual minority individual		1.59 (1.22-2.06)		<.01
Shop top sellers		—^c^		—
Shop by bottle size		—		—
Shop by brand		—		—
Shop by color		—		—
Price incentives				
Clearance or sale				
Age (y)		0.96 (0.94-0.99)		.01
Female		1.22 (1.06-1.41)		.02
Sexual minority individual		1.40 (1.12-1.75)		<.01
Deals (promo code, BOGOF^d^, and multipack)				
Female		1.22 (1.06-1.42)		.02
Direct discount				
Female		1.20 (1.03-1.41)		.04
Product curation				
New and trending				
Age (y)		0.93 (0.90-0.95)		<.01
Specs and features				
Bestsellers		1.19 (1.02-1.39)		.02
Female		1.19 (1.02-1.39)		.02
Starter kit				
Age (y)		0.97 (0.95-0.99)		.02
e-Cigarette use		0.87 (0.80-0.95)		<.01
Product rating or standard				
Authorized dealer				
Age (y)		1.05 (1.02-1.07)		<.01
Vape guide or blog (eg, Best of 2023)		—		—
Customer rating (≥3)		—		—
Age verification				
Enter email				
Age (y)		1.05 (1.02-1.08)		.01
e-Cigarette use		0.89 (0.81-0.98)		.04
Register or sign-in (each time)				
Age (y)		1.05 (1.02-1.08)		<.01
Female		0.65 (0.56-0.76)		<.01
Upload photo ID				
Age (y)		1.11 (1.08-1.13)		<.01
e-Cigarette use		0.88 (0.81-0.95)		<.01
Female		0.83 (0.72-0.95)		.02

a aOR: adjusted odds ratio.

b All reported *P* values were corrected for the false discovery rate (FDR), over the 18 items.

c Not applicable.

d BOGOF: buy-one-get-one free.

Finally, we examined susceptibility to use e-cigarettes as a function of participant characteristics and factors that may influence their decision to purchase. On the basis of participants with consistent responses, deals, new and trending products, a starter kit or option to shop by flavor were associated with higher likelihood of purchase (aOR 1.27‐1.67, 95% CI 1.07‐2.00; *P*<.01). In contrast, having to register or sign-in, enter an email ID, upload an ID or product specs and features was associated with lower susceptibility (aOR 0.71‐0.81, 95% CI 0.59‐0.97; *P*<.01). Model statistics are provided in Table 5. Corresponding results based on participants irrespective of the consistency of their responses are summarized in Table S3 in Multimedia Appendix 1. Finally, based on models that only examined associations between susceptibility and participant characteristics independently of their responses to marketing factors, female individuals were more likely to be susceptible to e-cigarette use (aOR 1.44, 95% CI 1.23‐1.68; *P*<.01). No other participant characteristic was associated with susceptibility.

**Table 5.** Statistics of models that assessed relationships between the susceptibility of participants who have never used e-cigarettes and the likelihood of purchasing based on individual marketing factors. Models are based on those with consistent, repeated responses.

Items	Susceptibility aOR^a^ (95% CI)		*P* value^b^
Product features			
Shop by flavor	1.67 (1.39-2.00)		<.01
Shop top sellers	—^c^		—
Shop by bottle size	—		—
Shop by brand	—		—
Shop by color	—		—
Price incentives			
Clearance or sale	—		—
Deals (promo code, BOGOF^d^, and multipack)	1.33 (1.11-1.59)		<.01
Direct discount	—		—
Product curation			
New and trending	1.28 (1.08-1.52)		<.01
Specs and features	0.81 (0.68-0.97)		.02
Bestsellers	—		—
Starter kit	1.27 (1.07-1.51)		.01
Product rating or standard			
Authorized dealer	—		—
Vape guide or blog (eg, Best of 2023)	—		—
Customer rating (>3)	—		—
Age verification			
Enter email	0.73 (0.60-0.88)		<.01
Register or sign-in (each time)	0.71 (0.59-0.85)		<.01
Upload photo ID	0.76 (0.64-0.90)		<.01

a aOR: adjusted odds ratio.

b All reported *P* values were corrected for the false discovery rate (FDR), over the 18 items.

c Not applicable.

d BOGOF: buy-one-get-one free.

## Discussion

### Principal Findings

In a large sample of 5326 adolescents and young adults, we have found that seeing specific content on e-cigarette retailer websites encourages adolescents and young adults to purchase e-cigarettes. Participants were more likely to purchase e-cigarettes if they saw keywords about flavor, price incentives (eg, clearance or sale or direct discounts), product curation or marketing including novelty and trendiness, or starter kits. Specifically, female and sexual minority individuals were more likely to respond positively to e-cigarette marketing and to purchase from websites if they saw price incentives and shopping by flavor. Female and sexual minority individuals were also less likely to purchase if they had to register or sign-in each time and upload a photo ID. Those who have never used e-cigarettes were more likely to purchase if they could shop by flavor or saw products marked as new and trending or starter kits. Furthermore, in comparison to those who had never used e-cigarettes, participants who had used them in the past 30 days were more likely to purchase if they were able to shop by flavor. Older adolescents and young adults were more likely to purchase from an authorized dealer, register or sign-in, provide an email ID, and upload a photo ID and less likely to respond to other content related to flavors, price, or product classification as trending.

Consistent with the latest research indicating that female individuals currently use e-cigarettes and are generally using substances at historically high rates compared with male individuals [22,23], our study found that female participants were more likely to purchase e-cigarettes if they saw marketing about flavors and price incentives. Prior studies have reported that approximately 75% of adolescents and young adults will not use e-cigarettes if they are not available in flavors, and young female adults find menthol, mint, or fruit flavors in e-cigarette advertisements more appealing than young male adults [4,24]. Potential reasons why female individuals may be using more include stress or self-medication for anxiety and depression (more common among female individuals) [25,26]. In fact, in our sample, approximately 60% of participants who reported browsing websites for e-cigarettes because they felt stressed were female. Female individuals may also likely use e-cigarettes if they are marketed for weight loss and improved socialization [27,28]. With increasing affordability of e-cigarettes [29] and 1 in 3 online e-cigarette advertisements including discounts [30], future research may examine why female and sexual minority individuals respond to price incentives more than their male and heterosexual counterparts, respectively.

Our findings related to sexual minorities being more likely to purchase e-cigarettes from online websites reflect a general trend that sexual or gender minority adolescents and young adults report greater engagement with online tobacco marketing than their heterosexual counterparts [31-33]. Given research that e-cigarette use is more likely among sexual minority individuals [34,35], there is a need to better understand potential factors influencing purchase and use behavior including identity-related stress and e-cigarette use as self-expression. Overall, adolescents and young adults who identify as female and as a sexual minority may benefit from tailored prevention efforts to make them more aware of e-cigarette marketing tactics, especially online.

The FDA and federal regulations, such as the Preventing Online Sales of E-Cigarettes to Children Act and the Prevent All Cigarette Trafficking Act (PACT), require online age and identity verification at the time of e-cigarette purchase without recommending specific methods to do so [36-38]. This study’s findings reveal that asking participants to register or sign-in each time, upload a photo ID, and enter their email ID may deter younger adolescents and young adults from purchasing online. Future research may assess whether these methods are effective across multibrand websites and e-cigarette company websites, since adolescents and young adults are acquiring e-cigarettes from both sources [39].

There are several plausible reasons why adolescents and young adults above the age of 21 years may be motivated to purchase from authorized dealers. Authorized dealers are more likely to verify age and require standardized payment methods such as credit cards; those aged below 21 years are more likely to use Venmo, home delivery apps, or purchase from neighborhood store websites and Amazon compared to those aged 21 to 40 years [39]. In addition, those who are aged 21 years and above may have been exposed to information about tetrahydrocannabinol e-cigarettes purchased from illicit online dealers being associated with e-cigarette, or vaping, product use–associated lung injury in 2020 [40] and therefore may prefer to access e-cigarettes from authorized sources.

Limited qualitative research shows that adolescents and young adults think starter kits are intended for those who have never used e-cigarettes and do not understand what a “starter kit” means (lower nicotine strength e-cigarette, smaller-size pack, or lower price) [18]. To grant Premarket Tobacco Marketing Authorizations, the FDA requires manufacturers to present risks and benefits for users and nonusers and provide evidence that the likelihood of initiation among those who have never used e-cigarettes is low [41]. Since starter kits influence adolescents and young adults who have never used or purchased e-cigarettes, the FDA may encourage manufacturers and e-cigarette retailer websites to (1) clearly communicate what a starter kit is in marketing material and packaging and (2) demonstrate evidence that their starter-kit line of e-cigarettes is not disproportionately used by youths and those who have never used.

### Limitations

This study had a few limitations. First, as a result of the random presentation of keywords, it is possible that some participants may not have seen all the 18 keywords at least once. Among those who saw keywords more than once, some had inconsistent responses. We addressed this issue by conducting 2 sets of analyses, including or excluding these participants, respectively, and identified similar patterns of associations. Second, we relied on self-reported data to an image-based prompt for a single brand, e-cigarette website, and sets of keywords. Therefore, we do not know whether participant responses would have been different if the image describing the choice task was of a multibrand e-cigarette website or an online store front managed by a brick-and-mortar vape shop. The impact of different keywords and content areas may differ based on additional nontextual elements such as fonts, colors, images, graphics, and size; we could not assess these effects independent of the content area in this study. Finally, we did not collect data on other factors (eg, cultural) that may influence adolescents and young adults to purchase e-cigarettes online.

### Conclusions

Female and sexual minority adolescents and young adults are more likely to purchase e-cigarettes from e-cigarette retailer websites if they see information related to flavors and price incentives. Those who have never used them are more susceptible to purchasing e-cigarettes if they see starter kits. Steps in age verification, such as asking to register or sign-in each time, upload a photo ID, and enter an email ID, could deter younger people (especially adolescents) from purchasing e-cigarettes from retailer websites. Online purchases could be reduced by improving awareness about flavors, promotional pricing and product curation, and starter kits among vulnerable populations, through prevention education and consistent age verification practices.

## Supplementary material

10.2196/75128 Multimedia Appendix 1 Survey question and additional results.

## ABBREVIATIONS

aOR: adjusted odds ratio
CHERRIES: Checklist for Reporting Results of Internet E-Surveys
e-cigarette: electronic cigarette
FDA: Food and Drug Administration
PACT: Preventing Online Sales of E-Cigarettes to Children Act and the Prevent All Cigarette Trafficking Act
