# Supplementary material for: Online e-Cigarette Retailers’ Use of Price Incentives and Product Features to Attract Adolescent and Young Adult Purchases: Cross-Sectional Choice-Based Study
Source: J Med Internet Res. 2025 Sep 29;27:e75128. doi: 10.2196/75128 (PMC12478966; doi:10.2196/75128)
Supplement: Multimedia Appendix 1 [file jmir-v27-e75128-s001.docx]

**Supplemental material**

**Figure S1. Screenshot of the survey question that preceded participants’ selection of keywords**


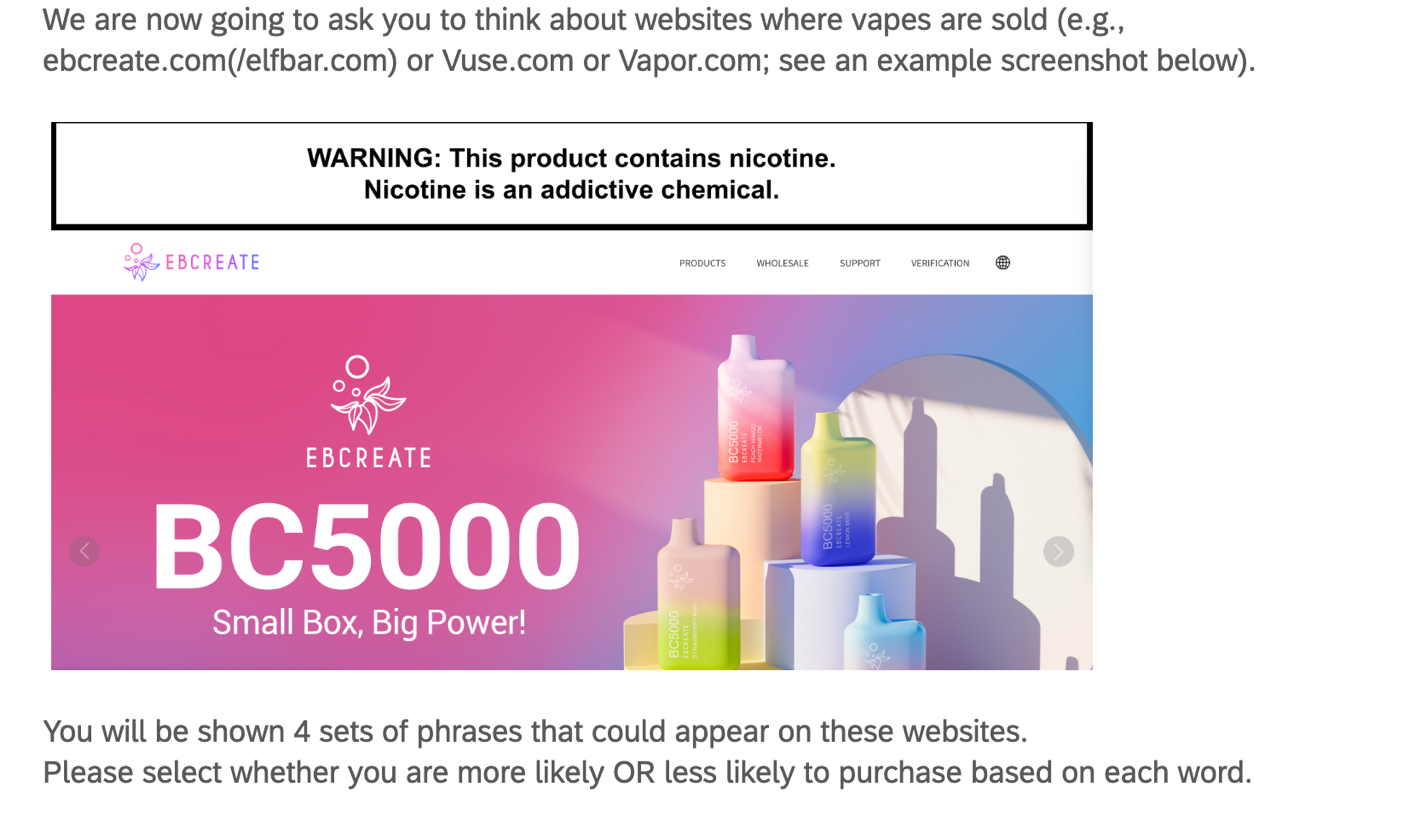


Screenshot of the Home Page of EBCreate.com

**Table S1.** Statistics of models testing associations between marketing factors and participant characteristics. Results are based on a sample that included all participants irrespective of consistency of repeated responses, and e-cigarette use (never used/vaped, used/vaped but not in the past 30 days, used/vaped in the past 30 days).

|  | **ITEMS** | **Parameter** | **aOR** | **95% CI** | **p-value^*^** |
| --- | --- | --- | --- | --- | --- |
| 1 | Authorized dealer | Age | 1.05 | [1.03, 1.07] | <0.01 |
| 2 | Bestsellers | None | | | |
| 3 | Clearance/Sale | Age | 0.97 | [0.95, 0.99] | 0.02 |
|  |  |  |  |  |  |
| 4 | Custom/ rating (3+) | None | | | |
| 5 | Deals (promo code, BOGOF, multi-pack) | Female | 1.19 | [1.06, 1.34] | 0.01 |
| 6 | Direct discount | Female  Sexual minorities | 1.24  1.22 | [1.10, 1.40]  [1.02, 1.48] | <0.01  0.04 |
|  |  |  |  |  |  |
| 7 | Enter email | Age | 1.03 | [1.01, 1.05] | 0.01 |
|  |  | Vaping history | 0.91 | [0.84, 0.98] | 0.04 |
|  |  | Female | 0.85 | [0.75, 0.97] | 0.02 |
|  |  |  |  |  |  |
| 8 | New and trending | Age | 0.95 | [0.93, 0.97] | <0.01 |
|  |  | Female | 1.15 | [1.02, 1.29] | 0.03 |
|  |  |  |  |  |  |
| 9 | Register/sign-in (each time) | Age | 1.03 | [1.01, 1.05] | <0.01 |
|  |  | Female | 0.68 | [0.60, 0.77] | <0.01 |
|  |  |  |  |  |  |
| 10 | Shop top sellers | None | | | |
| 11 | Shop by bottle size | None | | | |
| 12 | Shop by brand | None | | | |
| 13 | Shop by color |  | | | |
| 14 | Shop by flavor | Age | 0.97 | [0.95, 0.99] | 0.02 |
|  |  | Vaping history | 1.25 | [1.15, 1.35] | <0.01 |
|  |  | Female | 1.43 | [1.26, 1.61] | <0.01 |
|  |  |  |  |  |  |
| 15 | Specs and features | None | | | |
|  |  |  |  |  |  |
| 16 | Starter-kit | Age | 0.97 | [0.96, 0.99] | 0.02 |
|  |  | Vaping history | 0.90 | [0.83, 0.97] | 0.02 |
|  |  |  |  |  |  |
| 17 | Upload photo ID | Age | 1.09 | [1.07, 1.11] | <0.01 |
|  |  | Vaping history | 0.85 | [0.79, 0.92] | <0.01 |
|  |  | Female | 0.84 | [0.74, 0.94] | <0.01 |
|  |  |  |  |  |  |
| 18 | Vape guide or blog (e.g., best of 2023) |  | None |  |  |

*P-values have been corrected for the False Discovery Rate (over the 18 items).

**Table S2** Statistics of models testing associations between marketing factors and participant characteristics. Results are based on a sample that includes all participants irrespective of consistency of repeated responses, and compares those who had never vaped to those who had vaped in the last 30 days

|  | **ITEMS** | **Parameter** | **aOR** | **95% CI** | **p-value^*^** |
| --- | --- | --- | --- | --- | --- |
| 1 | Authorized dealer | Age | 1.05 | [1.03, 1.07] | <0.01 |
| 2 | Bestsellers | None | | | |
| 3 | Clearance/Sale | Age | 0.97 | [0.95, 0.99] | <0.01 |
|  |  | Sexual minorities | 1.36 | [1.12, 1.66] | <0.01 |
| 4 | Custom/ rating (3+) | None | | | |
| 5 | Deals (promo code, BOGOF, multi-pack) | Female | 1.20 | [1.06, 1.34] | 0.01 |
| 6 | Direct discount | Female  Sexual minorities | 1.22  1.23 | [1.07, 1.40]  [1.02, 1.52] | 0.03  0.04 |
|  |  |  |  |  |  |
| 7 | Enter email | Age | 1.03 | [1.01, 1.05] | 0.04 |
|  |  | Vaping history | 0.90 | [0.82, 0.98] | 0.04 |
|  |  | Female  Sexual minorities | 0.85  0.74 | [0.75, 0.97]  [0.60. 0.91] | 0.03  <0.01 |
|  |  |  |  |  |  |
| 8 | New and trending | Age | 0.95 | [0.93, 0.97] | <0.01 |
|  |  | Female | 1.17 | [1.03, 1.33] | 0.03 |
|  |  |  |  |  |  |
| 9 | Register/sign-in (each time) | Age | 1.04 | [1.02, 1.06] | <0.01 |
|  |  | Female | 0.81 | [0.59, 1.11] | <0.01 |
|  |  | Vaping history | 0.89 | [0.82, 0.98] | 0.03 |
|  |  |  |  |  |  |
| 10 | Shop top sellers | None | | | |
| 11 | Shop by bottle size | None | | | |
| 12 | Shop by brand | None | | | |
| 13 | Shop by color |  | | | |
| 14 | Shop by flavor | Vaping history | 1.21 | [1.11, 1.32] | <0.01 |
|  |  | Female  Sexual minorities | 1.40  1.42 | [1.22, 1.60]  [1.15, 1.75] | <0.01  <0.01 |
|  |  |  |  |  |  |
|  |  |  |  |  |  |
| 15 | Specs and features | None | | | |
|  |  |  |  |  |  |
| 16 | Starter-kit | Age | 0.97 | [0.94, 0.98] | <0.01 |
|  |  |  |  |  |  |
| 17 | Upload photo ID | Age | 1.10 | [1.07, 1.12] | <0.01 |
|  |  | Vaping history | 0.87 | [0.80, 0.94] | <0.01 |
|  |  | Female | 0.83 | [0.73, 0.94] | 0.01 |
|  |  |  |  |  |  |
| 18 | Vape guide or blog (e.g., best of 2023) |  |  |  |  |

**Table S3.** Susceptibility of those who have never used e-cigarettes. Models are based on participants irrespective of the consistency of their responses. Susceptibility was modeled as a binary variable.

|  | **ITEMS** | **Susceptibility aOR** | **95% CI** | | **p-value^*^** | |
| --- | --- | --- | --- | --- | --- | --- |
| 1 | Authorized dealer | - | | | | |
| 2 | Bestsellers | - | | | | |
| 3 | Clearance/Sale | - | | | | |
|  |  | - | | | | |
| 4 | Custom/ rating (3+) | - | | | | |
| 5 | Deals (promo code, BOGOF, multi-pack) | 1.24 | [1.02, 1.50] | | 0.03 | |
| 6 | Direct discount | - | | | | |
| 7 | Enter email | 0.71 | [0.58, 0.88] | | <0.01 | |
| 8 | New and trending | 1.22 | [1.04 ,1.48] | | 0.04 | |
| 9 | Register/sign-in (each time) | 0.76 | [0.62, 0.93] | | <0.01 | |
| 10 | Shop top sellers | - | | | | |
| 11 | Shop by bottle size | - | | | | |
| 12 | Shop by brand | - | | | | |
| 13 | Shop by color | - | | | | |
| 14 | Shop by flavor | 1.45 | [1.17, 1.78] | | <0.01 | |
| 15 | Specs and features | 0.81 | | [0.66, 0.98] | | 0.04 |
| 16 | Starter-kit | 1.23 | [1.03, 1.50] | | 0.04 | |
| 17 | Upload photo ID | 0.79 | 0.65, 0.95] | | 0.01 | |
| 18 | Vape guide or blog (e.g., best of 2023) | - | | | | |
